# The Impact of Medical Complications in Predicting the Rehabilitation Outcome of Patients With Disorders of Consciousness After Severe Traumatic Brain Injury

**DOI:** 10.3389/fnhum.2020.570544

**Published:** 2020-10-21

**Authors:** Lucia Francesca Lucca, Danilo Lofaro, Elio Leto, Maria Ursino, Stefania Rogano, Antonio Pileggi, Serafino Vulcano, Domenico Conforti, Paolo Tonin, Antonio Cerasa

**Affiliations:** ^1^S. Anna Institute, Crotone, Italy; ^2^Eng, deHealth Lab—DIMEG, UNICAL, Arcavata di Rende, Italy; ^3^San Giovanni di Dio Hospital, Crotone, Italy; ^4^Institute for Biomedical Research and Innovation (IRIB-CNR), Mangone, Italy

**Keywords:** severe traumatic brain injury (sTBI), medical complications, neurorehabiliation, machine learning, predictor factors

## Abstract

In this study, we sought to assess the predictors of outcome in patients with disorders of consciousness (DOC) after severe traumatic brain injury (TBI) during neurorehabilitation stay. In total, 96 patients with DOC (vegetative state, minimally conscious state, or emergence from minimally conscious state) were enrolled (69 males; mean age 43.6 ± 20.8 years) and the improvement of the degree of disability, as assessed by the Disability Rating Scale, was considered the main outcome measure. To define the best predictor, a series of demographical and clinical factors were modeled using a twofold approach: (1) logistic regression to evaluate a possible causal effect among variables; and (2) machine learning algorithms (ML), to define the best predictive model. Univariate analysis demonstrated that disability in DOC patients statistically decreased at the discharge with respect to admission. Genitourinary was the most frequent medical complication (MC) emerging during the neurorehabilitation period. The logistic model revealed that the total amount of MCs is a risk factor for lack of functional improvement. ML discloses that the most important prognostic factors are the respiratory and hepatic complications together with the presence of the upper gastrointestinal comorbidities. Our study provides new evidence on the most adverse short-term factors predicting a functional recovery in DOC patients after severe TBI. The occurrence of medical complications during neurorehabilitation stay should be considered to avoid poor outcomes.

## Introduction

Traumatic brain injury (TBI) is one of the leading causes of death and disability worldwide. Annually, over 2 million incidents are causing TBI and although research is continually accumulating to better understand the trajectory of clinical course, treatment options lag behind (Gaddam et al., 2015). Recovery from TBI is a complex process and severe brain injuries commonly result in a wide range of disorders of consciousness (DOC). This condition is characterized by high heterogeneity in clinical phenotypes and, mainly, in prognostic models (Langlois et al., 2006; Menon et al., 2010; Lasry et al., 2017) that contributed to disappointing results in several clinical trials (Menon, 2009).

One of the main nuisance factors affecting functional recovery from severe TBI is the presence of medical complications (MCs; Ganesh et al., 2013; Whyte et al., 2013; Pistoia et al., 2015). The presence of one or more MCs is associated with increased hospitalization time, worsened functional outcome, and increased mortality (Fu et al., 2015; Chan et al., 2017). Generally, these disorders are directly related to paroxysmal sympathetic hyperactivity (Lucca et al., 2019) or epileptic seizures (Pascarella et al., 2016). However, the presence of diabetes, ischemic heart disease, renal failure, and chronic obstructive pulmonary disease (Hansen et al., 2008) has also been commonly reported as risk factors for older TBI patients (Stocchetti et al., 2017).

Aging is one of the main factors increasing the level of heterogeneity in the clinical practice of TBI patients. Generally, owing to the aging of the population, a progressive increase in the incidence of TBI has been noted (Hamill et al., 2015; Haring et al., 2015; Gardner et al., 2018). As Gardner et al. (2018) and McIntyre et al. (2013) pointed out, elderly TBI patients differ from younger TBI in several factors, such as type of trauma, clinical course, and outcome. For instance, elderly TBIs are at greater risk of worse functional recovery than younger TBI patients (McIntyre et al., 2013). However, in literature, studies are showing that elderly TBI patients respond well to neurosurgical treatment and rehabilitation, suggesting that chronological age alone is not an adequate indicator of prognosis (McIntyre et al., 2013; Merzo et al., 2016). For this reason, it has been suggested that additional prognostic factors, such as MCs, should be considered when evaluating the clinical evolution of elderly TBI patients (Estraneo et al., 2018).

It is well known that MCs during the inpatient rehabilitation period strongly affect the functional outcome of TBI patients (Ganesh et al., 2013); also, younger age predicts improvement at 2–5 years after a traumatic event (Walker et al., 2018). Therefore, TBI patients with younger age and a low number of MCs should be promising phenotypes. However, the interaction between aging and MCs in TBI patients has poorly been investigated. The pre-injury factors influencing outcome after TBI are different as a function of age. Mathias and Wheaton (2015) demonstrated that elderly patients have less brain reserve and greater vulnerability that amplify brain damage and limit functional recovery. Moreover, Fu et al. (2015) found that the severity of lesions, the number of MCs, and older age are the most important predictors of intra-hospital mortality in these patients. Again, other clinical factors could unpredictably influence patients’ clinical evolution, such as the presence of ischemic or organic heart diseases (Pistoia et al., 2015) or altered brain activity (Sarà et al., 2011).

This longitudinal study is aimed at evaluating the predictive factors of functional outcome in DOC patients after severe TBI during the inpatient rehabilitation period. We sought to test whether an approach characterized by the employment of logistic regression and machine learning (ML) with random forest algorithm applied to several clinical factors may be able to predict the outcome as assessed by the Disability Rating Scale (DRS; Rappaport et al., 1982).

## Materials and Methods

### Subject Selection

**Figure 1:**
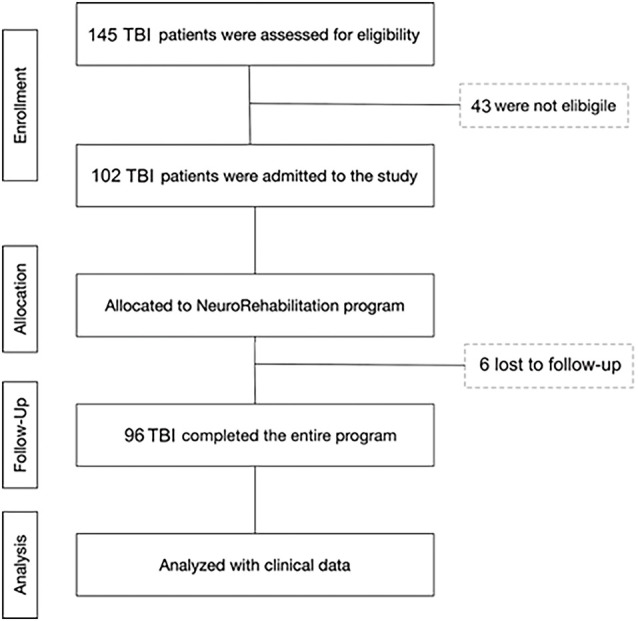
Flow diagram of participant recruitment and participation in the study.

All patients were consecutively admitted to the intensive rehabilitation unit (IRU) of the Institute S. Anna (Crotone, Italy) between January 2016 and December 2018. From an initial cohort of 145 TBI patients, we enrolled only those who fulfilled the following inclusion criteria: (1) severe TBI with Glasgow Coma Scale (GCS) score ≤8, identified based on medical records relative to the acute phase of the intensive care unit (ICU) period; (2) clinical diagnosis at IRU admission of vegetative state (VS), minimally consciousness state (MCS), or emersion according to standard diagnostic criteria (Giacino et al., 2002); (3) age ≥18 years; and (4) first admission to neurorehabilitation unit. Exclusion criteria were: (1) mild or moderate TBI; (2) the presence of a premorbid history of psychiatric disease or severe disability; and (3) ICU length of stay >90 days. From the initial group, 96 patients were selected and included in the final analysis phase. In total, 43 patients were not eligible because they were mild or moderate TBI, or were too young (<18 years), and six patients were not enrolled because they stayed for a long time in the ICU (>90 days; Figure 1).

All patients were transferred directly from the ICU after the medical and neurosurgery complications have been stabilized. Data from the acute hospital ICU are retrieved from patient files. The study was approved by the Ethical Committee of the University “Magna Graecia” of Catanzaro, according to the Helsinki Declaration. Written informed consent was obtained from the legal guardians of all patients.

### Procedure and Predictive Measures

This was a retrospective observational study. At admission and discharge, patients underwent a clinical evaluation of the consciousness state using the Coma Recovery Scale–revised (CRS-r; Giacino et al., 2002). The examination was performed by two neurologists and neuropsychologists with experience in disorders of consciousness who were blind to any other result. Patients who had already emerged from VS and MCS at admission underwent evaluation of cognitive functions. Moreover, to assess MCs and the disability at admission and during the follow-up, the modified Cumulative Illness Rating Scale (CIRS; Salvi et al., 2008) was used. The CIRS was selected because it allows a meaningful comparison of medical burden in individuals with variable and complex medical information and because it has been applied in a rehabilitation population (Holcomb et al., 2012). The CIRS is a 14-item rating scale used to indicate medical burden by rating impairment across 13 different organ systems (cardiac, hypertension, vascular, respiratory, eye/ear/nose/throat, upper gastrointestinal, lower gastrointestinal, hepatic, renal, other genitourinary, musculoskeletal, neurological, and endocrine-metabolic) as well as psychiatric/behavioral disturbances. Ratings for the “neurological” category were excluded for this study, thereby calculating a rating score of 0 (no impairment) to 4 (extremely severe impairment) for each item, and the total scores range from 0 to 52.

As predictive measures, we also considered additional demographic (age at the event, gender), imaging (Marshall classification of brain injury), and clinical variables. In particular, for the clinical domain, we considered cause of TBI, the presence of associated fractures (cranial, facial, vertebral, or extremities) or other trauma (thoracic, abdominal, spinal cord, or vascular), days in intensive care, GCS values at admission/discharge in ICU, Extended Glasgow Outcome Scale (GOSE) values at ICU discharge, CRS-r at admission to IRU, tracheostomy, respiration, feeding pathway, and MC measured by CIRS upon admission to IRU and during the follow-up period.

### Outcome Measures

The main outcome measure of this study was the evolution of the disability level as determined by the DRS (Rappaport et al., 1982), which was assessed at admission and discharge. The scale was developed for adults with moderate-to-severe TBI to track disability throughout recovery. In particular, not DRS raw score but transitions in the DRS disability category were considered and patients with stable or worse disability categories were compared with those who improved. The DRS consists of four categories (arousal and awareness, cognitive ability to handle self-care instructions, physical dependence on others, and psychosocial ability to work and perform daily functions).

### Clinical Treatment

All patients underwent a specialized rehabilitation program (Lucca et al., 2019), where they received a treatment of respiratory rehabilitation, active and passive mobilization, sitting posture conditioning, passive verticalization, training step pattern, and speech and cognitive therapies. VS and MCS patients also underwent unimodal sensory stimulation to promote specific cognitively mediated responses. The stimulation was intensively applied following a program including all sensorial fields (auditory, visual, tactile, olfactory; Riganello et al., 2013). As already demonstrated, the visual and auditory stimulations were used when the selected parameters of heart rate variability were in a specific range of intervals which maximize response (Riganello et al., 2016). Again, the treatment program included management of tone problems, autonomic disturbances, and other problems that are common in this population. If necessary due to spasticity or contractures, patients received therapy with injections of botulinum. Otherwise, patients who already emerged at admission from the vegetative state or the state of minimum consciousness underwent evaluation of superior cortical functions and conventional cognitive-behavioral rehabilitation treatment.

### Statistical Analysis

Statistical analyses were performed using R (version 3.5.3^1^). All data are presented as median (IQR) or count (%) as appropriate. Clinical characteristics (DRS and CRS-r) at admission and discharge from rehabilitation were compared using Wilcoxon signed-rank test with Pratt method.

Logistic regression was used to assess whether MCs were a risk factor independently from possible confounders for functional improvement failure. Hosmer–Lemeshow test was used to evaluate model goodness of fit, and no multicollinearity and influential observations were found.

Three different supervised ML algorithms, random forest, lasso regression, and support vector machine (SVM) with polynomial kernel, were used to develop predictive models of the outcome.

Random forest is an ensemble method based on merging the results of *n* classification tree. A classification tree is an algorithm that recursively split the data by finding the best cut-offs (among all the values of all the variables available) to create two subsets that are within them more similar in terms of class labels. Random forest, to improve the prediction performance of a single tree, build *n* decision tree each from a random subset of the observations and choosing a random subset of variables at each split. The new observations are then classified taking the class predicted by the majority of the *n* trees (Konukoglu and Glocker, 2020). Lasso (least absolute selection and shrinkage operator) regression analysis is a shrinkage and variable selection method originally proposed for linear regression models with a high number of variables. The goal of lasso regression is to obtain the subset of predictors that minimizes prediction error. The lasso does this by imposing a constraint on the regression model parameters (L1 regularization) that cause regression coefficients for some variables to shrink toward zero. Variables with a regression coefficient equal to zero after the shrinkage process are excluded from the model (feature selection). Variables with non-zero regression coefficients variables are most strongly associated with the response variable (Tibshirani, 1996). The idea of SVMs is to classify a set of data identified with two different class labels (binary classification) by finding, in the space of the variables, the hyperplanes that maximize the distance between data in the two classes. When this is not possible in the linear variables space (not linearly separable data), it is possible to apply a mathematical function called kernel (polynomial in the case of this analysis) to transform the space and linearly separate the data classes (Pisner and Schnyer, 2020).

The area under the ROC curve (AUC) was used to determine the best model’s performances. Fivefold cross-validation repeated five times was used to internally validate the models and select the best model parameters. Variables included in predictive analysis were age at event, sex, days in ICU, Marshall score assessed during ICU period, cause of TBI, fractures (cranial, facial, vertebral, extremities), trauma (thoracic, abdominal, spinal cord, or vascular), state of consciousness at IRU admission, CRS-r at IRU admission, breathing, feeding modalities, and MCs measured by CIRS upon admission to IRU and during the follow-up period. A variable importance measure was computed based on the mean decrease Gini index. For all tests, a *p* value < 0.05 was considered to be statistically significant.

## Results

### Clinical Characteristics at Admission and Discharge

From the initial cohort, 96 patients were selected for a retrospective observational evaluation of the rehabilitation program. Clinical and neurological variables collected in DOC patients at study entry are reported in Table 1. Endpoint measurements were obtained at a mean of 72 days post-onset (range 44–128).

**Table 1:** Clinical characteristics of the study cohort.

	TBI (*n* = 96)
Age (years)	43.6 ± 20.8 (18–77)
Sex (male)	69 (71.9%)
Length of stay ICU (days)	27.0 (20–35)
Length of stay IRU (days)	72.0 (43.8–128.2)
Cause of injury:	
Accidental fall	17 (17.7)
High-level fall	14 (14.6)
Car accident	13 (13.5)
Motorcycle accident	20 (20.8)
Pedestrian accident	7 (7.3)
Other	25 (26.0)
Marshall Score ICU (%)
I	0 (0.0)
II	35 (38.0)
III	18 (19.6)
IV	2 (2.2)
V	36 (39.1)
VI	1 (1.1)
Number of SAH (%)	44 (45.8)
Number of open head injuries (%)	4 (4.3)
Number of cranial fractures (%)	38 (39.6)
Extracranial injuries (%)	
Facial fractures	45 (46.9)
Extremities fractures	43 (44.8)
Vertebral fractures	23 (24.0)
Thoracic trauma	49 (51.0)
Abdominal trauma	17 (17.7)
Spinal cord Injury	2 (2.1)
Vascular trauma	4 (4.2)
GCS at ICU admission	4.0 (3.0–5.5)
GCS at ICU discharge	12.0 (9.0–13.2)
GOS at ICU discharge	3.0 (2.0–3.0)
CRS-r at IRU admission	23.0 (9.0–23.0)
Tracheostomy	59 (61.5)
Breath (%)	
Autonomous	75 (78.9)
Autonomous + O_2_	18 (18.9)
Mechanical	2 (2.1)
Feed (%)	
Oral	36 (37.9)
NG tube	41 (43.2)
PEG	18 (18.9)
Urinary catheter	91 (94.8)
Bedsore	31 (32.3)
Craniectomy	23 (24.0)

*ICU, intensive care unit; IRU, intensive rehabilitation unit; SAH, subarachnoid hemorrhage; GCS, Glasgow Coma Scale; GOS, Glasgow Outcome Scale; CRS-r, Coma Recovery Scale–revised; NG tube, nasogastric tube; PEG, percutaneous endoscopic gastrostomy*.

In Table 2, clinical and neurological variables collected in DOC patients at study entry and discharge are reported. At admission, 21% of patients were in VS, 22% in MCS, and 57% in emersion. At discharge from the neurorehabilitation unit, 76% had a full recovery of consciousness, whereas 12.5% remained in VS or MCS and 11.5% died. Univariate analysis demonstrated that disability was reduced in DOC patients at the discharge with respect to admission (Table 2).

**Table 2:** Clinical characteristics of the study cohort at admission and discharge from the rehabilitation unit.

	Admission	Discharge	*p*-level
Diagnosis (%)			0.040
Death	0 (0.0)	11 (11.5)	
Emersion	55 (57.3)	73 (76.0)	
MCS	21 (21.9)	8 (8.3)	
VS	20 (20.8)	4 (4.2)	
GOSE	3.0 (2.0–3.0)	3.0 (3.0–7.0)	<0.001
MCs (*n*)	5.0 (3.8–6.0)	2.0 (1.0–3.0)	<0.001
CRS-r	23 (9–23)	23 (21–23)	0.014
DRS	18 (16–21)	9 (5–15.2)	<0.0001

*MCS, minimally conscious state; VS, vegetative state; CRS-r, Coma Recovery Scale–revised; DRS, Disability Rating Scale; GOSE, Extended Glasgow Outcome Score; MC, medical complication*.

**Figure 2:**
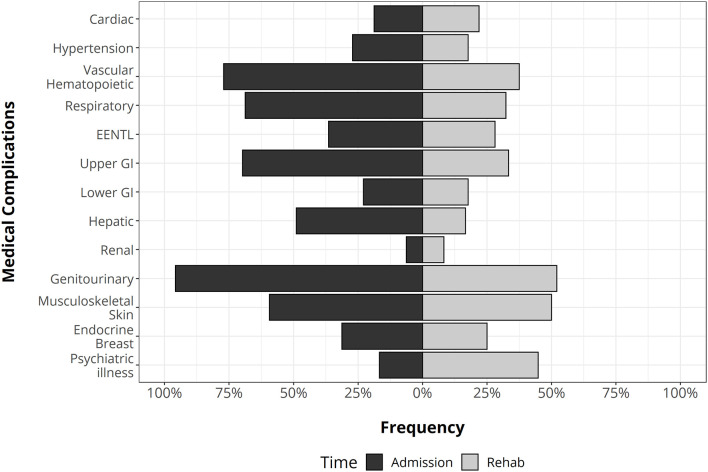
The distribution of medical complications in traumatic brain injury (TBI) patients at the admission (left side) and during rehabilitation stay (right side). GI, gastrointestinal apparatus. EENT complications: eye, ear, nose, throat, and larynx.

Overall, the distribution and the occurrence of MCs changed over time (Figure 2). At admission, genitourinary and vascular hematopoietic MCs were the most frequent, followed by respiratory and gastrointestinal. During the follow-up period, the most likely complications were genitourinary, gastrointestinal, musculoskeletal, and psychiatric illness, and the odds of MCs significantly decreased (*p*-level < 0.0001) with respect to admission. Indeed, the total number of MCs moved from 5 (range: 3.8–6) to 2 (range: 1–3).

### Predictive Models

A multivariable logistic regression model was employed to determine the risk of functional improvement failure as assessed by DRS scores. We adopted a twofold statistical approach to better characterize the nature of clinical factors. In the first data analysis, we performed logistic regression which allows us to evaluate the presence of a causal association between the number of MCs and the outcome, independently from age, sex, injury severity measured by Marshall CT score, and admission diagnosis. This statistical analysis demonstrated that among those variables, only the total amount of MCs was significantly associated with the DRS category worsening (OR: 1.63, 95% CI 1.06–2.53, *p* = 0.027; Table 3). As showed in Supplementary Figure 1, the total clinical outcome decreased as a function of age, but this effect did not emerge in regression analysis (1.025, 0.99–1.06, *p* = 0.13).

**Table 3:** Logistic model for association with functional improvement.

Variables	OR (95% CI)	*p*-level
Age (years)	1.025 (0.993–1.059)	0.131
Sex male (vs. female)	0.959 (0.26–3.544)	0.951
Marshall score at ICU admission III–VI (vs. I–II)	2.135 (0.527–8.649)	0.288
MCS at admission (vs. emerged)	1.585 (0.317–7.938)	0.575
VS at admission (vs. emerged)	1.564 (0.366–6.679)	0.546
Total number of MCs during IRU period	1.635 (1.058–2.526)	0.027

*MCS, minimally conscious state; VS, vegetative state; CRS-r, Coma Recovery Scale–revised; IRU, intensive rehabilitation unit; MC, medical complication*.

**Figure 3:**
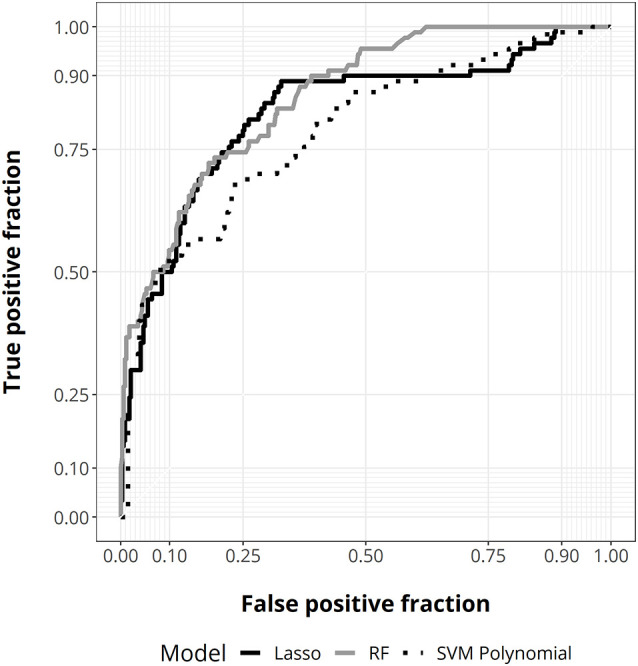
ROC curves for random forest (gray), lasso regression (solid black), and support vector machine (SVM) with polynomial kernel (dotted black).

Next, we performed an ML analysis using different algorithms to define the best predictive model and determine the importance value of factors in discriminating disability after treatment relative to their admission status. This analysis allows us to perform a predictive model following a non-linear approach. At internal validation, random forest showed the best predictive performance (AUC = 0.876, 95% CI 0.84–0.91; Figure 3, Supplementary Table 1).

**Figure 4:**
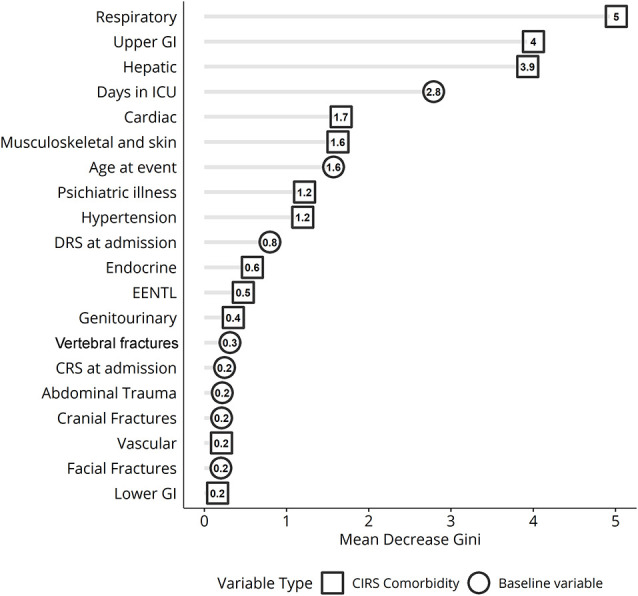
Variable importance ranking for random forest classification displaying the variables best discriminating patients based on Disability Rating Scale (DRS) scores measured before and after treatment in inpatient rehabilitation. ICU, intensive care unit; GI, gastrointestinal apparatus. EENT complications: eye, ear, nose, throat, and larynx.

Analysis of variable importance shows that the three most important clinical factors influencing the clinical outcome after the rehabilitation period are the respiratory, gastrointestinal, and hepatic MCs (Figure 4).

## Discussion

Within the inpatient rehabilitation period, the severe TBI group shows a moderate clinical improvement together with the appearance of at least one MC, which frequently occurs in severely brain-injured patients (Ganesh et al., 2013; Pistoia et al., 2015). Here, we used an ML approach to extract reliable markers of clinical changes. This advanced mathematical method reveals that the respiratory, gastrointestinal, and hepatic disorders are the most important factors influencing the prognosis of severe TBI patients, regardless of other demographical and clinical variables. We provide new evidence on the most adverse short-term factors leading to outcomes in DOC patients after severe TBI.

The outcome of TBI is strongly influenced by age. There are a lot of studies demonstrating that the elderly have a worse outcome after brain injury (Seidler et al., 2010; Kirkman et al., 2013; Peters et al., 2018), due to the greater likelihood to increase the occurrence of MCs, such as diabetes, ischemic heart disease, and chronic obstructive pulmonary disease, that may negatively impact outcome (Victorino et al., 2003; Taylor et al., 2017). Young age has been found by Estraneo et al. (2018) as one of the main predictors of the functional improvement in 194 patients with DoC followed for 6 months after the inpatient rehabilitation period. In the IMPACT multicentric study, investigating a very large sample (*n* = 8719) of TBI patients, Mushkudiani et al. (2007) reported that the most important predictive factor of the outcome was age, which showed an evident linear relationship with clinical status. Despite this kind of evidence, it is important to bear in mind that in the vast majority of these previous studies, the impact of demographical factors in predicting clinical outcome of TBI patients is not evaluated in conjunction with MCs. In other words, studies when age emerged as the main predictive factor did not often include MCs as an additional reliable predictor. Moreover, these studies used a linear statistical regression analysis approach, whereas we now proposed the employment of random forest to perform a predictive model following a non-linear approach. As shown in Figure 4, age is an import discriminator for the clinical status at discharge, but this is sixth behind four different MCs and the variable days in ICU. The discrepancy concerning previous literature may be easily explained by the fact that the increased number of MCs as well as the presence of problems in the respiratory, gastric, and hepatic domains is more frequent in the elderly patients. Thereby, the statistical variance related to age factor is in large part explained by some MCs which may drive regression analysis.

In the clinical management of TBI patients, the most frequent MCs are cardiorespiratory, pressure sores, gastrointestinal, infections, or the presence of multidrug-resistant bacteria (Whyte et al., 2013; Riganello et al., 2016; Scarponi et al., 2019). Generally, in TBI patients, the presence of more than three MCs during the inpatient rehabilitation is significantly associated with poorer clinical outcomes (Ganesh et al., 2013). Respiratory complications are one of the most frequent and noxious clinical events occurring during inpatient rehabilitation period. As already explained by Estraneo et al. (2018), these are most often caused by pulmonary infections, related to the presence of tracheostomy and systemic immune suppression. This kind of complication has shown to be the main cause of death in individuals with moderate or severe TBI (Greenwald et al., 2015). A study of 224 severe TBI patients admitted to ICU found that the most common cause of death is respiratory infections or severe respiratory failure. Overall, respiratory diseases and arrhythmias without organic heart diseases are the most frequent MCs and the strongest predictors of missed recovery of consciousness and functional improvement (Pistoia et al., 2015). Our regression findings are perfectly in agreement with this latter evidence. Gastrointestinal complications, together with paroxysmal sympathetic hyperactivity, have been demonstrated to be the most likely severe risk factor in DoC patients (Taylor et al., 2017). Remarkably, the third discriminatory factor identified by the ML algorithm was hepatic complications. This clinical problem is sparsely reported in this patient. Although surprising, this result may be explained by a very recent work that highlights the importance of hepatic pathology in the various symptomology associated with TBI. In particular, Nizamutdinov et al. (2017) demonstrated that TBI induces pathological alterations including elevation of hepatic acute, phase proteins, hepatic inflammation, together with alterations of the bile acid receptors and transporters in the liver and hypothalamus. These authors proposed that these findings are compatible with previous evidence in stroke models, where it was demonstrated that alterations in the bile acid system may increase apoptosis and its neuroprotective role after brain injury (Rodrigues et al., 2002). For this reason, we believe that our findings can open a new window for the evaluation of hepatic complications in TBI patients which until now has been neglected.

## Limitations

Some possible limitations of our data need to be addressed. First of all, we did not consider the impact of pre-existing clinical characteristics emerging during the ICU period on the final outcome, such as elevated intracranial pressure and cranial surgery. Although this kind of information is needed, a recent study did not reveal their usefulness in a predictive prognostic model of long-term functional outcomes for severe TBI patients (Walker et al., 2018). As concerns complications occurring in the IRU period, we did not evaluate the different impacts of single or recurrent seizures on outcomes, which is known as one of the predictors of poor outcome (Estraneo et al., 2018). This is dependent on the fact that the occurrence of seizures is immediately pharmacologically treated during the inpatient rehabilitation period. Similarly, we did not report evidence on the impact of some other important clinical complications occurring in this kind of patients, such as paroxysmal sympathetic hyperactivity and infections (Estraneo et al., 2018). Next, the monocentric nature of the present study together with the short time outcomes (2–3 months) might limit its generalization. Although part of our results are similar to those reported in long-term longitudinal studies (Ganesh et al., 2013; Riganello et al., 2013; Whyte et al., 2013; Estraneo et al., 2018), we are aware that this study needs further evaluation before translating to clinical practice. Finally, we did not evaluate if MCs occurred during the ICU period. The pre-existing comorbidities should be assessed because they might favor the vulnerability to secondary neurologic and medical complications.

## Conclusions

Rehabilitation of DOC patients after severe TBI in the early weeks after injury is critical and constitutes a significant multidisciplinary challenge constantly evolving. Here, we provide useful information about the predictive factors of functional outcomes to guide clinicians’ therapeutical intervention and prevention efforts (Scarponi et al., 2019). Our statistical approach stresses the role of MCs as the predictor of poor clinical outcome. These findings confirm that the inpatient rehabilitation period is critical for this kind of patients, who increasingly require monitoring over time by experienced clinicians with the knowledge of preventing and managing clinical problems.

## Data Availability Statement

The raw data supporting the conclusions of this article will be made available by the authors, without undue reservation.

## Ethics Statement

The studies involving human participants were reviewed and approved by Central Area Regione Calabria of Catanzaro, Italy. The patients/participants provided their written informed consent to participate in this study.

## Author Contributions

Statistical analysis was done by DL. Study design was done by LL, EL, and MU. Drafting the article was done by LL and AC. Clinical data collection was made by SR and AP. Literature search, data interpretation, and revising the article were done by PT, DC, and SV. All authors contributed to the article and approved the submitted version.

## Conflict of Interest

The authors declare that the research was conducted in the absence of any commercial or financial relationships that could be construed as a potential conflict of interest.

The handling editor declared a past co-authorship with one of the authors PT.

## Footnotes

^1^https://www.R-project.org/
