# Supplementary material for: The Impact of Medical Complications in Predicting the Rehabilitation Outcome of Patients With Disorders of Consciousness After Severe Traumatic Brain Injury
Source: Front Hum Neurosci. 2020 Oct 21;14:570544. doi: 10.3389/fnhum.2020.570544 (PMC7641612; doi:10.3389/fnhum.2020.570544)
Supplement: Supplementary file 1 [file Table_1.DOCX]

**ONLINE SUPPLEMENTARY MATERIAL**

**The impact of medical complications in predicting clinical outcome of patients with severe traumatic brain injury: a machine learning study**

Lucca Lucia Francesca, Lofaro Danilo, Leto Elio, Ursino Maria, Rogano Stefania, Pileggi Antonio, Vulcano Serafino, Conforti Domenico, Tonin Paolo, Cerasa Antonio.

**Results**

***Aging effect on TBI-related disability***

In order to evaluate the simple relationship between age, comorbidities and disability in TBI patients during neurorehabilitation stay, a univariate correlation analysis (*r*’s of Person) was performed. As shown in Figure S1, Admission-Discharge DRS delta values was positively related to age (r = 0.537, 95%CI 0.375-0.667, p < 0.0001), as well as, the number of comorbidities (r = 0.579, 95%CI 0.426-0.699, p < 0.0001). In other words, elderly TBI patients are generally characterized by decreased disability recovery.


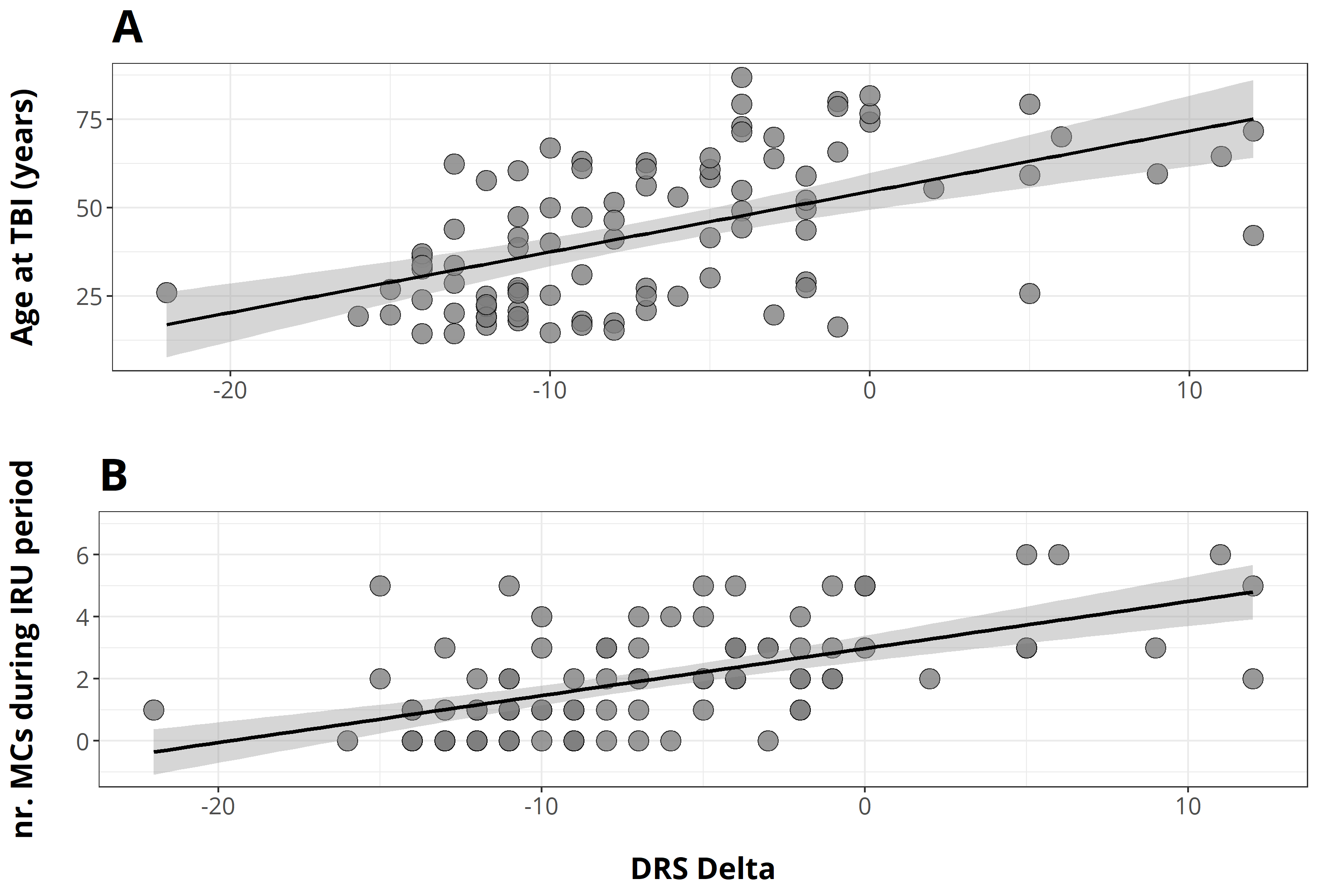


**Figure S1.** Admission - Discharge DRS delta vs Age (A) and nr. of severe comorbidities (MCIRS >= 2; B)

**Table S1.** Predictive performance of the tested Machine Learning models.

| **Models** | **AUC (95%CI)** | **Sensitivity** | **Specificity** | **PPV** | **NPV** |
| --- | --- | --- | --- | --- | --- |
| RF | 0.876 (0.838-0.914) | 0.773 | 0.789 | 0.932 | 0.483 |
| Lasso | 0.817 (0.762-0.872) | 0.678 | 0.889 | 0.958 | 0.426 |
| SVM Poly | 0.811 (0.762-0.86) | 0.624 | 0.856 | 0.941 | 0.379 |

*AUC: Area Under ROC Curve; PPV: Positive Predictive Value; NPV: Negative Predicted Value; RF: Random Forest; SVM: Support Vector Machines.*
